# The Influence of Technostress on Cyberslacking of College Students in Technology-Enhanced Learning: Mediating Effects of Deficient Self-Control and Burnout

**DOI:** 10.3390/ijerph191811800

**Published:** 2022-09-19

**Authors:** Xinghua Li, Dehua Liu

**Affiliations:** 1 School of Educational Science, Hunan Normal University, Changsha 410081, China; 2 School of Business, Hunan Institute of Technology, Hengyang 421002, China

**Keywords:** technostress, cyberslacking, deficient self-control, burnout, chain mediating effect

## Abstract

College students frequently experience technostress and engage in cyberslacking whilst participating in technology-enhanced learning (TEL). This research aimed to investigate the influence mechanism of technostress on college students’ cyberslacking. This research recruited 634 students from two Chinese colleges to complete a web-based questionnaire adapted from previous research. Structural equation modelling was adopted and the research results showed that: in TEL (1) college students’ technostress significantly and positively affected cyberslacking; (2) deficient self-control partially mediated college students’ technostress and cyberslacking; (3) burnout partially mediated college students’ technostress and cyberslacking; and (4) deficient self-control and burnout played a chain mediating role between college students’ technostress and cyberslacking. These findings improve our understanding of the influence college students’ technostress has on cyberslacking in TEL, and several suggestions to reduce college students’ cyberslacking in TEL are proposed.

## 1. Introduction

In recent years, colleges have accelerated the integration of information and communication technology (ICT) into students’ learning. Technology-enhanced learning (TEL), such as Massive Open Online Courses (MOOCs), flipped classrooms, and blended classrooms, is widely adopted to increase the convenience and efficiency of students’ learning [1]. While TEL provides a number of advantages for students’ learning, some disadvantages are also associated with the technology [2]. One of such disadvantages is the use of the Internet by students for personal purposes during lectures [2], which has been described as cyberslacking in previous studies [2,3,4].

Cyberslacking (or cyberloafing) refers to using devices and the Internet for off-task purposes during work or lectures [5]. The educational literature reports that college students’ cyberslacking behaviors include checking emails, playing online games, using social networking websites, reading news, and shopping online [6,7,8]. Students’ cyberslacking behaviors may lead to negative consequences, such as distraction [2], smartphone addiction [9], and poor academic performance [10]. With easy access to mobile devices and wireless Internet in TEL, cyberslacking is increasingly noticeable among college students [2,11]. A self-reported survey showed that students spent 40% of their online time on cyberslacking (e.g., managing e-mail and surfing the Web) while at school [12]. The rate is likely to increase as ICT is increasingly integrated into education [13,14]. Given the high prevalence of cyberslacking and negative consequences, it is important to investigate the key factors that influence students’ cyberslacking when they are engaging in TEL. An improved understanding of these factors may lead to the development of appropriate interventions.

Previous studies have revealed that stress is an important antecedent of cyberslacking [15,16,17], and technostress may significantly predict white-collar employees’ cyberslacking [18]. Technostress may occur when individuals’ skills and abilities are poorly aligned with the demands of ICT [19], and it frequently leads to a lack of attention [20], which in turn results in increasing cyberslacking [2,18]. In TEL, various learning applications and mobile devices could cause information overload and life invasion for college students, which may in turn induce technostress [21] and cyberslacking [8]. Thus, considering the significant correlations between technostress and cyberslacking, it is important to explore the influence mechanism of technostress on college students’ cyberslacking.

According to the strength model of self-control [22], people may engage in cyberslacking when they lack self-control [23], while some empirical studies have shown that technostress may result in deficient self-control [24], which may in turn induce cyberslacking [25]. Alternatively, the stress-strain-outcome model [26] suggests that cyberslacking may also be an outcome of stress and burnout [17,27]. According to previous empirical studies, technostress could lead to burnout [1], and burnout may induce cyberslacking [28]. Furthermore, deficient self-control and burnout are not isolated phenomena, and deficient self-control is positively correlated with burnout [29]. Thus, we posit that deficient self-control and burnout play a chain mediating role between technostress and cyberslacking.

Based on the strength model of self-control and the stress-strain-outcome model, this study aimed to explore the effect of technostress on college students’ cyberslacking in TEL, and the potentially mediating effects of deficient self-control and burnout on technostress and cyberslacking. The results will assist us to develop a better understanding of the important factors that may impact college students’ cyberslacking in TEL and subsequently inform the development of effective interventions to reduce students’ cyberslacking. 

## 2. Literature Review and Development of Hypotheses

### 2.1. Technostress and Cyberslacking

Technostress refers to maladaptation resulting from a person’s inability to adapt to the requirements of using ICT [30]. While previous studies have primarily focused on employees’ technostress [31,32,33,34], researchers have more recently found that college students are also likely to suffer from technostress when they are engaging in TEL [1,19,35,36].

Three categories of technostress have been identified in TEL: techno-overload, techno-complexity, and techno-invasion [21]. Techno-overload describes the situation where students are confronted with large amounts of information created by various learning applications and are compelled to learn quickly to manage multitasking. Techno-complexity refers to the situation where the complexity of learning applications compels students to devote more time to adapt to technology. Techno-invasion relates to the situation where students can be contacted anytime and anywhere in TEL, which may blur the boundary between learning and one’s personal life. Under any of these conditions, college students may suffer from technostress [21], which may in turn, generate emotional reactions such as frustration, depression, and exhaustion [21,37], and behavioral reactions such as reduced engagement in TEL, and using the Internet for socialization or entertainment [21,38,39]. 

Existing studies have previously confirmed the relationship between technostress and cyberslacking, and it has been reported that employees’ technostress is positively associated with the use of social media, a typical example of cyberslacking [40]. In addition, Güğerçin [18] demonstrated that white-collar employees’ technostress positively predicted their cyberslacking behaviors. However, whether this relationship exists among college students when engaging in TEL has yet to be tested which was the aim of the current study. Specifically, this study proposed that H1:

**H1.**  *Technostress leads to college students’ cyberslacking in TEL*.

### 2.2. The Mediating Role of Deficient Self-Control between Technostress and Cyberslacking

Self-control is the ability of people to maintain conscious control over their actions against impulses, habits, or automated responses [22], and deficient self-control is defined as a state in which conscious self-control is relatively diminished [41]. Some studies have reported that constant stress may reduce individuals’ self-control [42,43,44]. In the TEL context, students are required to exert self-control to cope with technostress [24], which is caused by cognitive overload, the occupation of personal time, and unexpected errors from technology [19]. It has also been reported that technostress may indirectly impair college students’ self-control [45]. Hence, the current study posits that technostress may positively influence college students’ deficient self-control when they are engaging in TEL.

According to the strength model of self-control [22], an individual’s ability to exercise self-control is a limited resource. Any activity consuming strength resources, such as emotion management, mental control, and decision making, can lead to the depletion of self-control. When an individual exerts excessive self-control resources on a task, he/she might be unable to complete a subsequent task that also requires self-control. Furthermore, previous studies have shown that people with deficient self-control are more likely to be involved in cyberslacking [23,46]. Thus, the present study inferred that college students’ deficient self-control may induce cyberslacking when they are engaging in TEL. 

Furthermore, a previous study revealed that deficient self-control played a critical mediating role between technostress and college students’ academic performance [24]. Thus, this study hypothesized H2:

**H2.**  *Deficient self-control mediates the effect of technostress on college students’ cyberslacking when they are engaging in TEL*.

### 2.3. The Mediating Role of Burnout between Technostress and Cyberslacking

Burnout was first proposed by Freudenberger [47], and typical symptoms include physical exhaustion, fatigue, and psychological drainage [28]. Researchers have primarily focused on the effect of burnout on employees [27,34]. More recently, researchers have researched the effect of burnout on college students engaging in TEL [1,45]. 

According to the stress-strain-outcome model [26], students under constant stress may suffer burnout, which will, in turn, induce adverse outcomes (e.g., cyberslacking behaviors). Previous studies have reported that technostress is positively associated with emotional exhaustion and burnout among college students [21,48]. In addition, people with higher levels of work or academic burnout are more likely to engage in cyberslacking behaviors [28]. Moreover, previous empirical research has demonstrated that burnout plays a mediating role between perceived stress and cyberslacking [17], and thus this study hypothesized H3:

**H3.**  *Burnout mediates the effect of technostress on college students’ cyberslacking when they are engaging in TEL*.

### 2.4. The Chain Mediating Role of Deficient Self-Control and Burnout between Technostress and Cyberslacking

The strength model of self-control [22] suggests that when people’s self-control resources are diminished, they may enter a state of ego depletion, which manifests in fatigue, exhaustion, and burnout. The relationship between self-control and burnout has been documented in previous empirical studies. For example, Seibert et al. [29] demonstrated that students’ self-control was negatively correlated to school burnout, while Love et al. [49] found that college students with deficient self-control were more vulnerable to burnout. Therefore, it can be inferred that deficient self-control may lead to the burnout in college students.

In TEL, the growing demands of ICT can lead to increasing technostress among students [1], which may deplete their self-control resources, in turn leading to burnout and cyberslacking. Furthermore, it has been demonstrated that stress indirectly influences individuals’ mobile phone addiction via the chain mediating role of self-control and burnout [50]. As cyberslacking is associated with smartphone addiction [9], it can be inferred that deficient self-control and burnout may play a chain mediating role between technostress and cyberslacking. Therefore, this study hypothesized H4:

**H4.**  *Deficient self-control and burnout play a chain mediating role between technostress and cyberslacking*.

**Figure 1:**
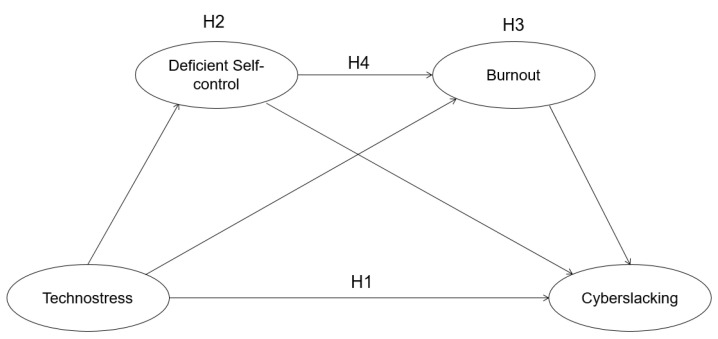
Hypothetical model of the relationship between technostress, deficient self-control, burnout and cyberslacking.

This study explored the influence of technostress on cyberslacking, both independently and secondarily via its influence on deficient self-control and burnout (Figure 1). 

## 3. Methods

### 3.1. Design and Procedure

In our research, we used a survey method to explore the effect of technostress on college students’ cyberslacking during TEL. Considering that the participants of this study were Chinese college students, we translated the original scales, which were English-version, into Chinese following the back-translation method. We employed a researcher majoring in English to translate the Chinese-version scales back into English. And then, the Chinese-version scales were modified until there were no significant discrepancies between the back-translation and the original scales in English. As for the content validity of the scales, two researchers who are familiar with our research reviewed the scales for relevance and clarity. After a pretest was conducted with 12 college students, redundant and ambiguous items were shortened and refined for better clarity.

Currently, Chinese colleges strongly promote technology-enhanced learning, and almost all college students have attended courses in a TEL format, such as flipped classrooms and MOOCs [19]. Participants of this research were students studying at a Chinese first-class college and a general college. We invited participants who had experienced TEL through Wechat, a popular social media tool of China, and data were collected through the online questionnaire platform Wenjuanxing, from 25 March to 10 April 2022. An informed consent process was conducted before the survey. Participants were informed that their participation was voluntary and anonymous, and they could choose to stop the survey at any time. 

### 3.2. Instrument Development

Four scales were developed to assess technostress, deficient self-control, burnout and cyberslacking in this study. All were derived from existing scales and internal consistency was assessed via factor analysis. 

#### 3.2.1. The Technostress Scale

The technostress scale (TS) was designed to measure the level of technostress experienced by students when they were engaging in TEL. The scale was adapted from Wang et al. [19] and included four items; for example, “I feel stressed to adapt to technology-enhanced learning.” The scale was answered using a five-point Likert scale, with responses ranging from “strongly disagree” to “strongly agree”. The Cronbach’s alpha coefficients of this scale was 0.921.

#### 3.2.2. The Deficient Self-Control Scale

The deficient self-control scale (DSC) measured the level of individuals’ deficiency in self-control. The items for this study were selected from the Deficient Self-Control Scale [24,41] and were modified to fit the TEL context; for example, “I feel my Internet use is out of control, in technology-enhanced learning.” The scale was answered using a five-point Likert scale, with responses ranging from “strongly disagree” to “strongly agree”, and higher scores indicated higher deficiency in self-control. The Cronbach’s alpha coefficients of this scale was 0.896.

#### 3.2.3. The Burnout Scale

The burnout scale (BO) measured the level of students’ burnout in TEL. The scale was adapted from Wang et al. [19] and included four items, for example, “I feel overwhelmed due to technology-enhanced learning.” The scale was answered using a five-point Likert scale, with responses ranging from “strongly disagree” to “strongly agree”. The Cronbach’s alpha coefficients of this scale was 0.872.

#### 3.2.4. The Cyberslacking Scale

The cyberslacking scale (CS) measured the frequency of students’ cyberslacking behaviors when participating in TEL. The scale was adapted from Gökçearslan et al. [9] and Metin et al. [51], and included four items, for example, “I do online shopping during studying hours, in the technology-enhanced learning.” The scale was answered using a five-point Likert scale, with responses ranging from “strongly disagree” to “strongly agree”. The Cronbach’s alpha coefficients of this scale was 0.909.

### 3.3. Data Analysis

Data were analyzed using SPSS 21.0 and AMOS 24.0. SPSS 21.0 was used to generate descriptive statistics, to assess the reliability of the questionnaires, and to conduct correlational analyses. This study used AMOS 24.0 to conduct the following analyses: (1) Confirmatory factor analysis; (2) Common method variance test; (3) Structural equation modelling (SEM); and (4) Mediating effects test using a non-parametric percentile bootstrap method.

## 4. Results

A total of 634 responses were recorded, and 604 were retained after the exclusion of invalid questionnaires. Participants included 301 (49.8%) males and 303 (50.2%) females; 343 (56.8%) first-year students, 99 (16.4%) second-year students, 147 (24.3%) third-year students, and 15 (2.5%) fourth-year students.

### 4.1. Common Method Variance Test

Confirmatory factor analysis was performed to evaluate the common method variance (CMV) problem. The confirmatory factor analysis fit indexes of the single-factor model and the multi-factor model are presented in Table 1. The chi-square value of the multi-factor model (*χ*^2^ = 211.769) was significantly lower than the single-factor model (*χ*^2^ = 636.774), suggesting that the two models were significantly different (∆*χ*^2^ = 425.004, ∆*df* = 16, *p* < 0.001), and indicating that there were no severe CMV problems [52].

### 4.2. The Measurement Model

This study evaluated the measurement model using the following indicators: standardized factor loadings, Cronbach’s alpha coefficients (α), component reliability (CR), average variance extracted (AVE), discriminant validity, and the fitness of the model. 

As Table 2 illustrates, factor loadings of all instrument items were above 0.50. In addition, Cronbach’s alpha coefficients for all four instruments were above 0.80 (TS: 0.921; DSC: 0.872; BO: 0.896; CS: 0.909), indicating acceptable internal consistency [53]. Results also showed that all CR values were greater than 0.70, and all AVE values were greater than 0.50 (Table 2). All values fit the criteria [54], indicating adequate convergent validity. 

This study conducted Pearson correlation analysis to assess the correlation between variables. Table 3 illustrates that technostress and deficient self-control had a significant and positive correlation (r = 0.417, *p* < 0.001); technostress and burnout had a significant and positive correlation (r = 0.323, *p* < 0.001); technostress and cyberslacking had a significant and positive correlation (r = 0.369, *p* < 0.001); deficient self-control and burnout had a significant and positive correlation (r = 0.536, *p* < 0.001); deficient self-control and cyberslacking had a significant and positive correlation (r = 0.523, *p* < 0.001); burnout and cyberslacking had a significant and positive correlation (r = 0.684, *p* < 0.001). In addition, the square root of each AVE was higher than the correlation coefficients with other constructs (Table 3), suggesting acceptable discriminatory validity [55]. 

This study used CFA to test the measurement model fit. The model fit indexes were *χ*^2^/*df* (chi-square/degree of freedom) = 3.728, TLI (Tucker-Lewis index) = 0.956, CFI (comparative fit index) = 0.964, NFI (normed fit index) = 0.952, RMSEA (root mean square error of approximation) = 0.067 (Table 4), indicating that the fitness of the measurement model was acceptable [56].

### 4.3. The Structural Model

#### 4.3.1. Technostress and Cyberslacking

SEM was used to assess the main effects of technostress on cyberslacking. The model fit indexes were: *χ*^2^/*df* = 3.364, TLI = 0.981, CFI = 0.987, NFI = 0.982, and RMSEA = 0.062, indicating acceptable fit [56]. The findings revealed that college students’ technostress significantly and positively predicted their cyberslacking (β = 0.37, *p* < 0.001), supporting H1.

#### 4.3.2. Chain Mediating Effect of Deficient Self-Control and Burnout between Technostress and Cyberslacking

**Figure 2:**
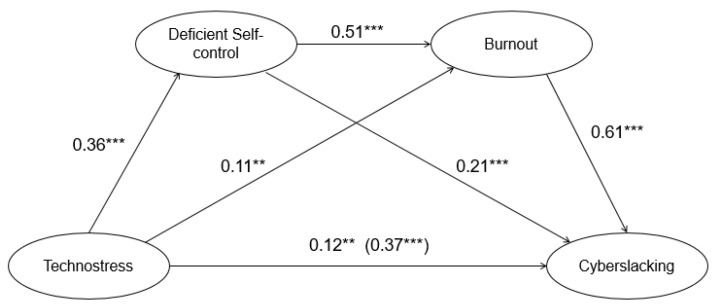
Structural equation model of the influence of technostress, deficient self-control and burnout on cyberslacking. ** *p* < 0.01; *** *p* < 0.001.

This study adopted SEM to construct and validate the chain mediating model (Figure 2). The model fit indexes were as follows: *χ*^2^/*df* = 3.728, TLI = 0.956, CFI = 0.964, NFI = 0.952, RMSEA = 0.067 (Table 4), indicating the chain mediating model fits well [56].

The chain mediating model (Figure 2) comprises three pathways that contribute to the indirect effect of technostress on cyberslacking. A bias-corrected percentile bootstrap method was used to evaluate the indirect effect with 5000 bootstrap resamples.

In the first instance, the estimated value of the indirect effect of technostress on cyberslacking through deficient self-control was 0.076 (β = 0.076, *p* < 0.01). Furthermore, the 95% confidence interval (CI) was 0.029–0.134 (Table 5), suggesting that deficient self-control was a significant mediator in the relationship between technostress and cyberslacking, supporting H2.

Secondly, the estimated value of the indirect effect of technostress on cyberslacking through burnout was 0.067 (β = 0.067, *p* < 0.05). Furthermore, the 95% CI was 0.010–0.128 (Table 5), suggesting that burnout significantly mediated the relationship between technostress and cyberslacking, supporting H3.

Finally, the estimated value of the indirect effect of technostress on cyberslacking through deficient self-control and burnout was 0.113 (β = 0.113, *p* < 0.01). Furthermore, the 95% CI was 0.073–0.172 (Table 5), suggesting that deficient self-control and burnout were significant mediators in the relationship between technostress and cyberslacking, supporting H4.

## 5. Discussion

This research posited technostress significantly and positively impacted college students’ cyberslacking when they are engaging in TEL, and deficient self-control and burnout mediated the relationship between technostress and cyberslacking. The findings of this study supported all the hypotheses.

Study findings validated H1 and confirmed that college students’ technostress significantly and positively impacted cyberslacking. This result is consistent with those of previous studies, which revealed that people with higher levels of technostress were more likely to engage in cyberslacking [18,40,57], possibly because technostress might cause students to divert their attention from learning activities to cyberslacking activities [20]. While previous studies regarding the relationship between technostress and cyberslacking have mainly focused on employees in the workplace, the current study extends discussion of this relationship to college students in TEL, and highlights the negative impacts of ICT on college students’ learning. 

The findings also validated H2, i.e., that deficient self-control plays a mediating role between technostress and cyberslacking among college students. The result is consistent with previous studies that have shown that technostress may lead to deficient self-control [24,58], and deficient self-control induces cyberslacking [9,25,59]. This finding also supports the strength model of self-control [22], which suggests that individuals may fail in the tasks requiring self-control, after having used excessive self-control resources in previous tasks. The results indicate that students who perceive technostress may be more likely to deplete their self-control resources, which in turn, induces cyberslacking. It confirms that deficient self-control plays an important mediating role between technostress and cyberslacking. 

H3, i.e., that burnout mediates technostress and cyberslacking among college students, is also supported by the findings, and is consistent with those of previous studies. For instance, it has been shown that technostress may lead to burnout [1,17,27,34,60], and burnout may induce individuals’ cyberslacking [17,61]. This finding also supports the stress-strain-outcome model [26], which reveals that stress might lead to strain, and induce adverse outcomes. It suggests that students’ technostress may induce burnout, which, in turn, causes them to engage in cyberslacking, and confirms that burnout plays a crucial mediating role between technostress and cyberslacking.

Finally, the findings validated H4, i.e., that college students’ deficient self-control and burnout mediated the relationship between technostress and cyberslacking—a result that is consistent with those of previous studies. For instance, researchers found that individuals’ deficient self-control positively predicted burnout [49,62] while Qin [50] demonstrated that college students’ self-control and burnout played a chain mediating role between perceived stress and mobile phone addiction. The results also support the strength model of self-control [22], which suggests that constant exertion of self-control may lead to ego depletion, which in turn causes burnout and leads to cyberslacking. This finding is an important contribution of this research, which revealed that technostress among college students not only has significant direct effects on cyberslacking, but also has indirect effects on cyberslacking through deficient self-control and burnout. 

## 6. Implications

These findings suggest some possible measures to address technostress and cyberslacking. In the first instance, educators and students should be more concerned about the role of technostress, as the COVID-19 Pandemic required remote teaching and TEL will be increasingly used in colleges in the future [63]. TEL should be considered to be a double-edged sword with both benefits and shortcomings. While TEL provides efficiencies to students, TEL may induce technostress and cyberslacking. According to previous studies, there are several ways in which educators can guide students to cope appropriately with technostress, such as providing technical support [1], enhancing students’ information literacy [27], and undertaking mindfulness training [34].

Secondly, educators and students should focus on enhancing students’ capacity for self-control. Given that this study found deficient self-control is a critical factor in explaining how technostress leads to cyberslacking, educators should incorporate self-control in training projects to improve students’ capacity for self-control.

Finally, educators and students should take measures to avoid students’ burnout. As burnout is an essential antecedent to cyberslacking, educators should help students cope with technostress and burnout effectively through training or counseling [1]. When college students suffer from technostress and burnout in TEL, they need to take a break or turn to their educators for guidance.

## 7. Limitations

The current study has certain limitations. First, the study participants are all from Hunan Province, China, which may limit the generalizability of the findings. To address this limitation, future researchers could consider selecting a wider sample from a wider range of national and cultural backgrounds. Second, this study explored the mediating role of deficient self-control and burnout between technostress and cyberslacking through a cross-sectional study, and hence it was not possible to demonstrate a causal relationship between the two variables. The use of longitudinal studies and time series analyses would overcome this limitation. Third, the survey of cyberslacking in this study used a self-report scale with no objective data on cyberslacking. This could be remedied in future research by collecting objective data on cyberslacking including the frequency and amount of time spent on cyberslacking.

## 8. Conclusions

This study found that technostress, deficient self-control, and burnout were important factors influencing college students’ cyberslacking. The results demonstrated that (1) technostress affected college students’ cyberslacking directly; (2) deficient self-control exerted a partial mediating effect on technostress and college students’ cyberslacking; (3) burnout exerted a partial mediating effect on technostress and college students’ cyberslacking; and (4) deficient self-control and burnout played a chain mediating role between technostress and college students’ cyberslacking. This study contributes to the knowledge base regarding technostress and cyberslacking and demonstrated the relationship between technostress, deficient self-control, burnout, and cyberslacking among college students. Several possible interventions were suggested to reduce college students’ cyberslacking by alleviating technostress, strengthening self-control, and avoiding burnout when they are engaging in TEL. 

## Figures and Tables

**Table 1:** Comparison of single-factor and multi-factor models.

Model	*χ* ^2^	*df*	*χ* ^2^ */df*	∆*χ*^2^	∆*df*	*p*
Single-factor mode	636.774	98	6.498	425.004	16	0.000
Multi-factor model	211.769	82	2.583			

**Table 2:** Results of the Confirmatory Factor Analysis.

Latent Variable	Measurement Variable	Mean	Std. Dev.	Factor Loadings	α	CR	AVE
TS	TS1	2.770	0.038	0.834	0.921	0.923	0.751
	TS2			0.917			
	TS3			0.920			
	TS4			0.788			
DSC	DSC1	2.910	0.038	0.663	0.896	0.881	0.655
	DSC2			0.734			
	DSC3			0.939			
	DSC4			0.870			
BO	BO1	2.820	0.037	0.688	0.872	0.877	0.643
	BO2			0.751			
	BO3			0.895			
	BO4			0.857			
CS	CS1	3.150	0.380	0.815	0.909	0.910	0.716
	CS2			0.845			
	CS3			0.895			
	CS4			0.827			

TS = technostress, DSC = deficient self-control, BO = burnout, CS = cyberslacking.

**Table 3:** Discriminant validity of the research instruments.

Scale	TS	DSC	BO	CS
TS	0.867			
DSC	0.417 ***	0.809		
BO	0.323 ***	0.536 ***	0.802	
CS	0.369 ***	0.523 ***	0.684 ***	0.846

*** *p* < 0.001. Bolded fonts are AVE square root values.

**Table 4:** The goodness of fit indices of the measurement model and the research model.

Model	*χ* ^2^	*χ*^2^/*df*	TLI	CFI	NFI	RMSEA
Measurement model	361.576 (0.000)	3.728	0.956	0.964	0.952	0.067
Research model	361.576 (0.000)	3.728	0.956	0.964	0.952	0.067
Recommended criteria	*p* > 0.05	<5.0	>0.90	>0.90	>0.90	<0.08

**Table 5:** Results of the bootstrap analysis.

Path	Point Estimation	Product of Coef.		bias-Corrected	
		SE	Z	Lower	Upper
Technostress→Deficient Self-Control→Cyberslacking	0.076	0.027	2.861	0.029	0.134
Technostress→Burnout→Cyberslacking	0.067	0.030	2.263	0.010	0.128
Technostress→Deficient Self-Control→Burnout→Cyberslacking	0.113	0.025	4.579	0.073	0.172
Total effect	0.375	0.056	6.649	0.267	0.489

## Data Availability

Data is available upon request to the corresponding author.
